# Distinct diagnostic and prognostic values of Glypicans gene expression in patients with hepatocellular carcinoma

**DOI:** 10.1186/s12885-021-08104-z

**Published:** 2021-04-26

**Authors:** Jian-Yao Wang, Xiang-Kun Wang, Guang-Zhi Zhu, Xin Zhou, Jun Yao, Xiao-Peng Ma, Bin Wang, Tao Peng

**Affiliations:** 1 Department of Hepatobiliary Surgery, The First Affiliated Hospital of Guangxi Medical University, Shuang Yong Road 6#, Nanning, 530021 Guangxi Zhuang Autonomous Region People’s Republic of China; 2 Department of General Surgery, Shenzhen Children’s Hospital, Yi Tian Road 7019#, Shenzhen, 518026 Guangdong Province People’s Republic of China; 3 Department of Gastroenterology, Jinan University of Second Clinical Medical Sciences, Shenzhen Municipal People’s Hospital, Dong Men Bei Road 1017#, Shenzhen, 518020 Guangdong Province People’s Republic of China

**Keywords:** HCC, Diagnosis, Prognosis, Glypicans, Bioinformatics analysis

## Abstract

**Backgroud:**

In our current work, we aimed to investigate the expressions of glypican (GPC) family genes at the mRNA level and assess their prognostic significances in patients with hepatocellular carcinoma (HCC).

**Methods:**

The pathological roles of GPC family genes were examined using bioinformatics analysis. The diagnostic values of GPC genes were explored with the Gene Expression Profiling Interactive Analysis. Moreover, the mRNA expression and prognostic values of GPC genes were assessed via the KM plotter database.

**Results:**

Our data showed that the expression of GPC-3 was dramatically increased in the liver tumor tissue. Moreover, the expressions of the other five GPC family members were not significantly different between the tumor and normal liver tissues (*P* > 0.05). Furthermore, the up-regulation of GPC-1 at the mRNA level was dramatically correlated to the reduced overall survival (OS) for all HCC patients (hazard ratio = 2.03, 95% confidence intervals =1.44–2.87, *P* = 4.1e-05) compared with its low-expression group. Besides, the prognosis of the Caucasians was related to most GPC family genes, while the prognosis of the Asian race was only related to the expression of GPC-2. Besides, for pathological factors, including stage, grade, AJCC, and vascular invasion, the higher the pathological grade and vascular invasiveness, the lower the expression levels of GPC family genes (*P* < 0.05). Finally, the expression levels of GPC-1, 2, and 3 in the hepatitis group were related to the poor prognosis of HCC in the risk factor (alcohol consumption and hepatitis) subgroup (*P* < 0.05).

**Conclusions:**

Our findings indicated that GPC-3 was dysregulated in HCC compared with paracancerous tissues. The expression of GPC-1 could be used as a potent predictive index for the general prognosis of HCC. The pathology, patients, and risk factors might affect the prognostic value of GPC family genes in HCC.

**Supplementary Information:**

The online version contains supplementary material available at 10.1186/s12885-021-08104-z.

## Background

Hepatic cancer is more frequently diagnosed in males compared with females, and a dramatically increasing number of patients with liver cancer have been reported in recent years. Most of the primary liver cancer is diagnosed as HCC [1]. HCC ranks the second leading cause of tumor-associated mortality worldwide, accounting for more than 90% of all deaths from primary HCC. Although a great deal of effort has been made on HCC [2], the mechanism underlying the pathogenesis of HCC remains largely unexplored.

Glypicans (GPCs) are a group of heparan sulfate (HS) proteoglycans, which are identified to be associated with the exocytoplasmic surface of the plasma membrane through a glycosyl-phosphatidylinositol anchor. The GPC family consists of six members, namely GPC-1 ~ 6 [3–5]. These members show high expressions on the cell surface and in the extracellular matrix, which function mainly as mediators of growth factor signaling pathway. Recent investigations have shown that some of GPC genes are dysregulated in the pathogenesis of various tumors, which may probably participate in tumorigenesis, and these detectable proteins in the blood can be used as latent clinical indicators [6–10].

Like other tumors, the tumorigenesis of HCC can also be attributed to genetic and environmental origins. Moreover, dysregulated genes involved in the pathogenesis of tumors are the most prospective source of diagnostic and prognostic indices.

However, an in-depth investigation is required to comprehensively assess the diagnostic and prognostic values of GPC genes in HCC. In the present study, we aimed to explore the diagnostic and prognostic values of GPC family genes in HCC patients according to information derived from publicly accessible databases and bioinformatics assay.

## Methods

### Bioinformatics analysis of GPC family genes

Gene Ontology (GO) and Kyoto Encyclopedia of Genes and Genomes (KEGG) enrichment analyses of GPCs were performed using the database for Annotation, Visualization and Integrated Discovery [11–13] (DAVID)v6.8 (accessed October 27, 2019; https://david.ncifcrf.gov/home.jsp;). The gene-gene interaction (GGI) network was established using the gene multiple association network integration algorithms [14–16] (GeneMANIA; http://www.genemania.org/; accessed October 27, 2019), and STRING [17, 18] (Search Tool for the Retrieval of Interacting Genes/Proteins) (STRINGv.10.0; https://string-db.org/;accessed October 27, 2019) was employed to build protein-protein interaction (PPI) network.

### Correlation analysis and assessment of the diagnostic value

The database of Gene Expression Profiling Interactive Analysis [19](GEPIA) was used (accessed October 27, 2019; http://gepia.cancer-pku.cn/index.html) to probe the differential expressions of GPCs.

As a newly established interactive web server, GEPIA can evaluate the RNA sequencing data from the TCGA and GTEx projects [20], including 9736 tumors and 8587 normal samples, using a standard processing pipeline.

### Construction of prognostic signature

The detailed information of GPC family genes was submitted to the online database Kaplan–Meier plotter [21, 22] (KM plotter; http://kmplot.com/analysis/index.php?p=service&cancer=liver_rnaseq), which can investigate the effects of 54,000 genes on overall survival (OS) in 21 types of cancers. The largest dataset includes liver, breast, ovarian, lung, and gastric cancers. The miRNA subsystems include 11,000 samples from 20 different types of cancer. The database includes gene chip and RNA-seq data sources for different databases, such as Gene Expression Omnibus (GEO), European Genome-phenome Archive (EGA), and The Cancer Genome Atlas (TCGA). Therefore, the KM plotter has been widely accepted for assessing the clinical impacts of individual genes on the survival of cancer patients, including HCC. In our current study, the gene expressions of GPC family genes and their prognostic values in HCC patients were explored via the KM plotter database.

The KM survival plots were drawn according to the medians of clinical parameters, including pathology factor (stage, grade, AJCC, vascular invasion), patient factor (sex, race, sorafenib treatment), and risk factor (alcohol consumption and hepatitis virus). Based on the above-mentioned clinical parameters, the patients were assigned to different subgroups.

### Statistical analysis

SPSS v.22.0 software (IBM Corp, Armonk, NY, USA) was employed to conduct the statistical analysis. The data consisted of hazard ratio (HR), survival plot, 95% confidence intervals (CI), and log-rank. A *p* value of less than 0.05 was considered statistically significant.

## Results

### Bioinformatics analysis of GPC family genes

**Fig. 1:**
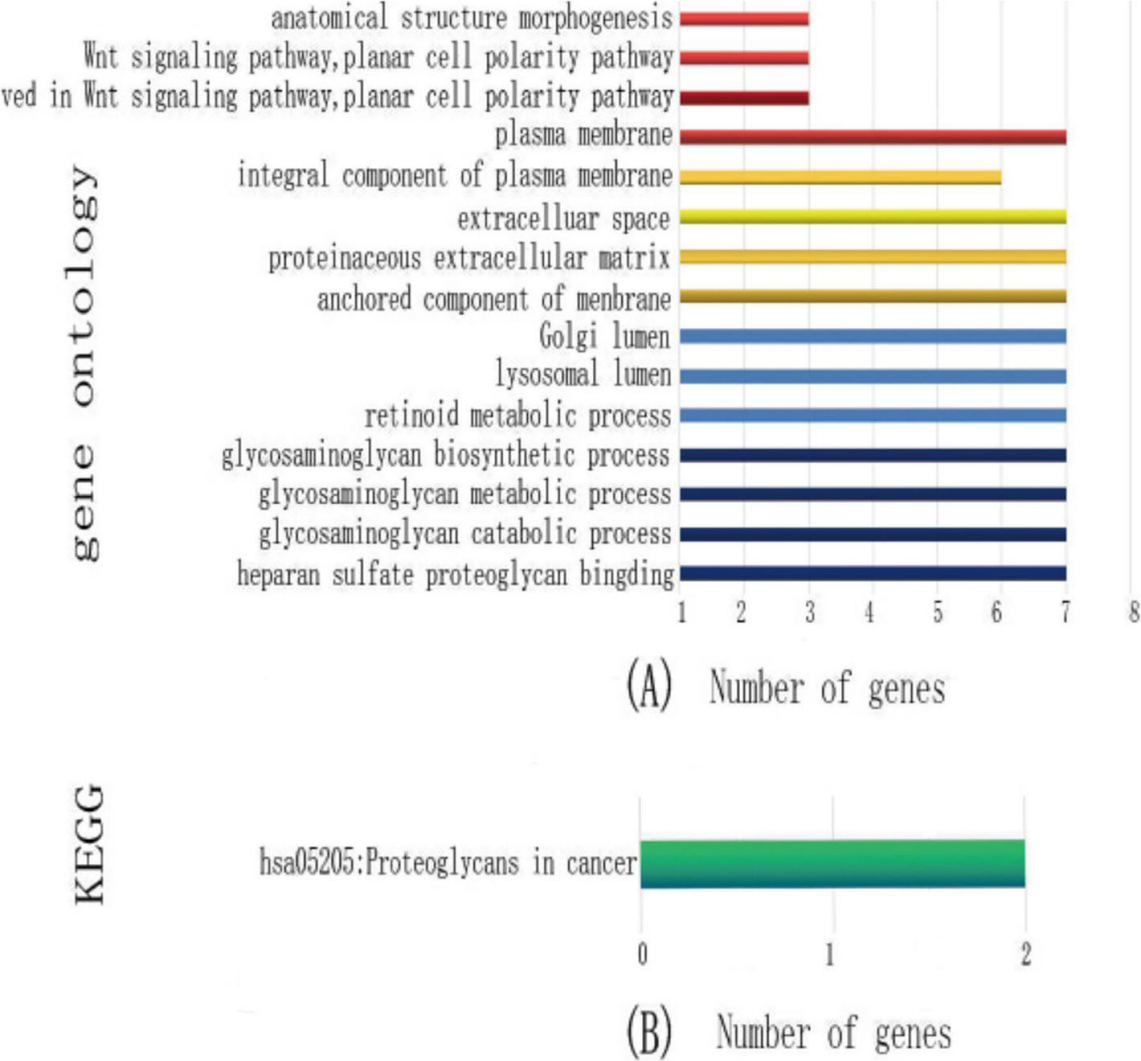
GO and KEGG analyses of GPC genes. **a** GO pathway assay of GPC genes. **b** KEGG pathway assay of GPC genes

Figure 1a shows the results of GO analysis. The functions of GPC family genes included anatomical structure morphogenesis, Wnt signaling pathway, planar cell polarity pathway, co-receptor activity involved in Wnt signaling pathway, planar cell polarity pathway, plasma membrane, integral component of plasma membrane, extracellular space, proteinaceous extracellular matrix, anchored component of membrane; Golgi lumen, lysosomal lumen, retinoid metabolic process, glycosaminoglycan biosynthetic process, glycosaminoglycan catabolic process, and HS proteoglycan binding. However, the KEGG pathway analysis using DAVID suggested that the functions of GPC family genes were involved in hsa05205: proteoglycans in cancer (Fig. 1b).

**Fig. 2:**
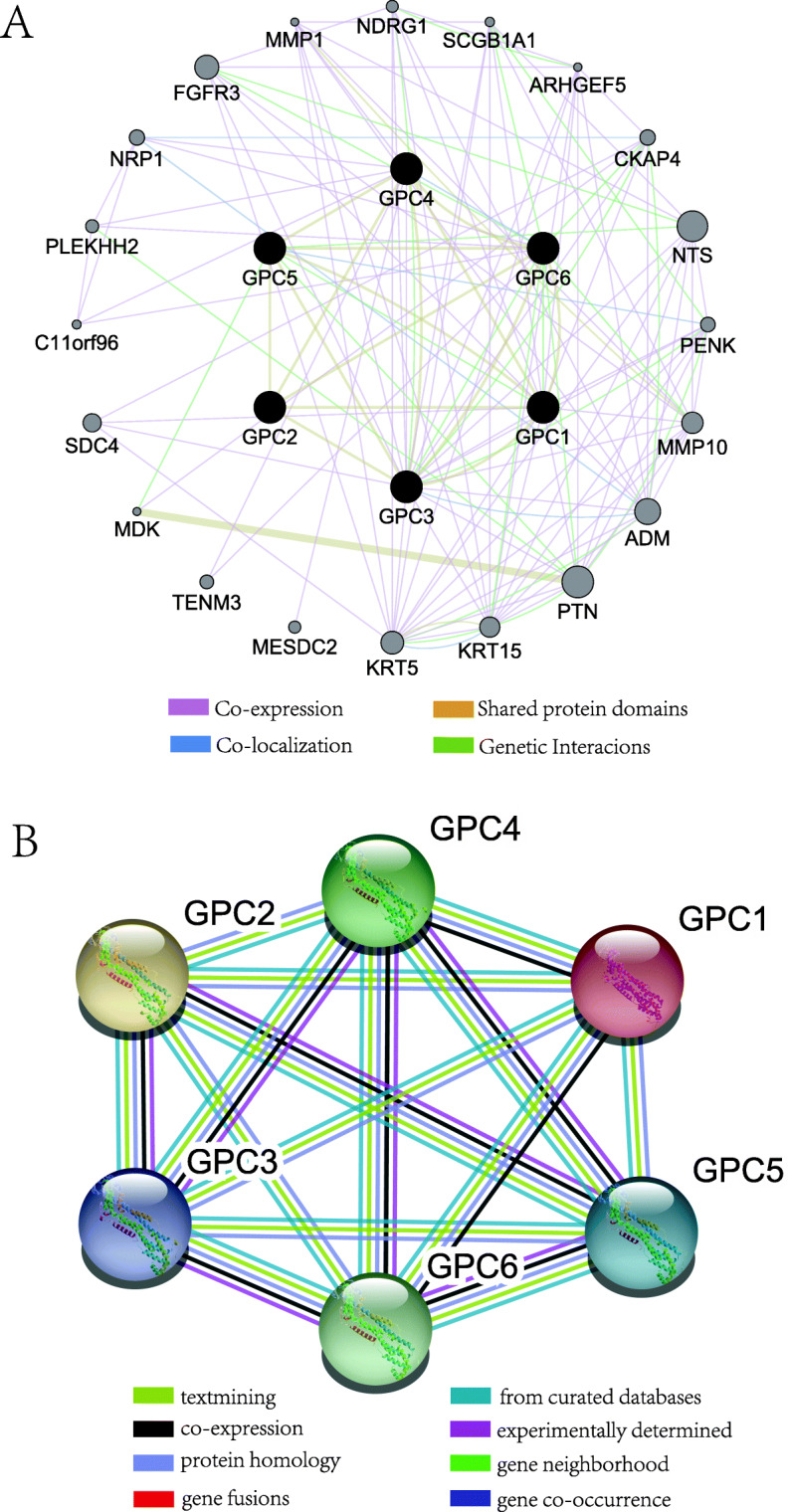
GGI and PPI networks of GPC genes. **a** Gene multiple association network integration algorithm. **b** PPI networks

GeneMANIA was used to conduct a correlation analysis of GPC family members at the gene level, which revealed relationships in pathways, shared protein domains, co-localization, and co-expression between GPC1 to GPC6. A complex network was constructed with the GPC family genes and other related genes (Fig. 2a). GGI network suggested that the GPC family genes possessed a strong protein homology and co-expression pattern with each other. STRING analysis was performed to identify the interactions of GPC gene family members at the protein level. The PPI network indicated that GPC family genes contacted with each other directly or indirectly. GPC3 was shown to interact with GPC1, GPC2, GPC4, GPC5, and GPC6 in regards to gene co-occurrence, text-mining, co-expression, and protein homology. The GPC3 gene closely related to other GPC family genes was in the central position. There were relationships among GPC2, GPC4, GPC6, and GPC3 in texting, protein homology, and gene co-occurrence. Besides, there were relationships in text-mining and protein homology among GPC1, GPC5, and GPC3.

### Correlation analysis and assessment of the diagnostic value

**Fig. 3:**
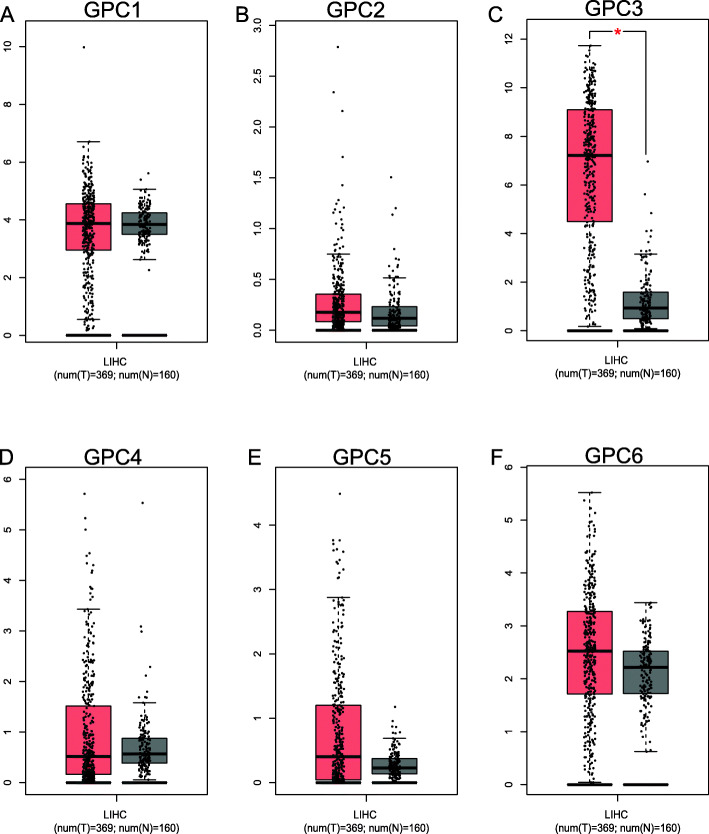
Expression distribution of GPC family genes in HCC using the GEPIA. **a**-**f** GPC-1 ~ GPC-6

In the present study, we compared the expressions of GPC family genes between liver tumor tissues and paracancerous tissues using the GEPIA online tool. Our data showed that GPC-3 was significantly up-regulated in the liver tumor tissues (Fig. 3c). Moreover, the expressions of GPC-1, GPC-2, GPC-4, GPC-5, and GPC-6 were not significantly different between tumor and normal liver tissues (Fig. 3a, b, d, e, f).

### Correlation analysis between the expressions of GPC family genes at the mRNA level and OS

**Fig. 4:**
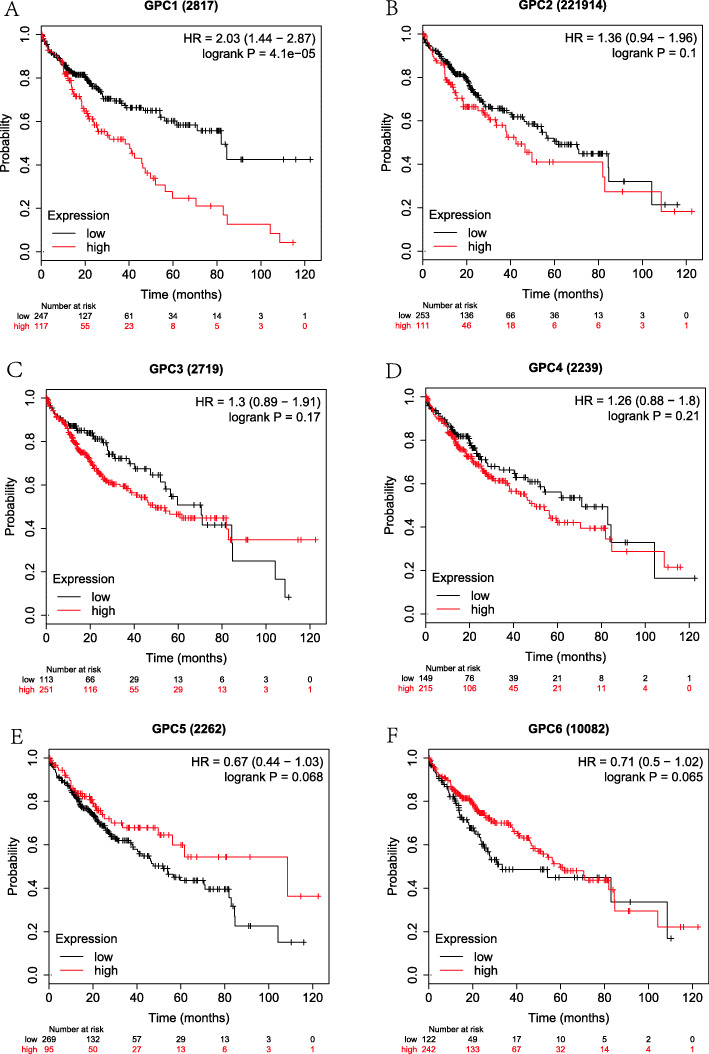
The prognostic value of the mRNA expressions of GPC genes in all HCC patients using KM plotter tool. **a**-**f** GPC-1 ~ GPC-6.(*n* = 364)

**Fig. 5:**
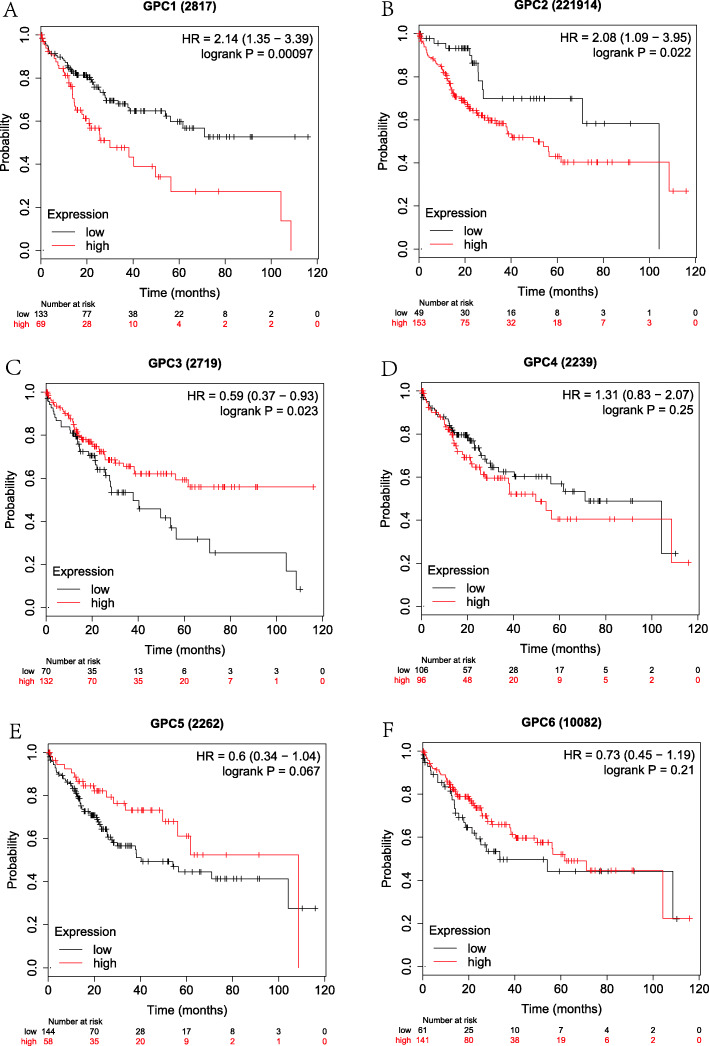
The prognostic value of the mRNA expressions of GPC genes in non-alcohol consumption HCC patients using KM plotter tool. **a**-**f** GPC-1 ~ GPC-6.(*n* = 202)

**Fig. 6:**
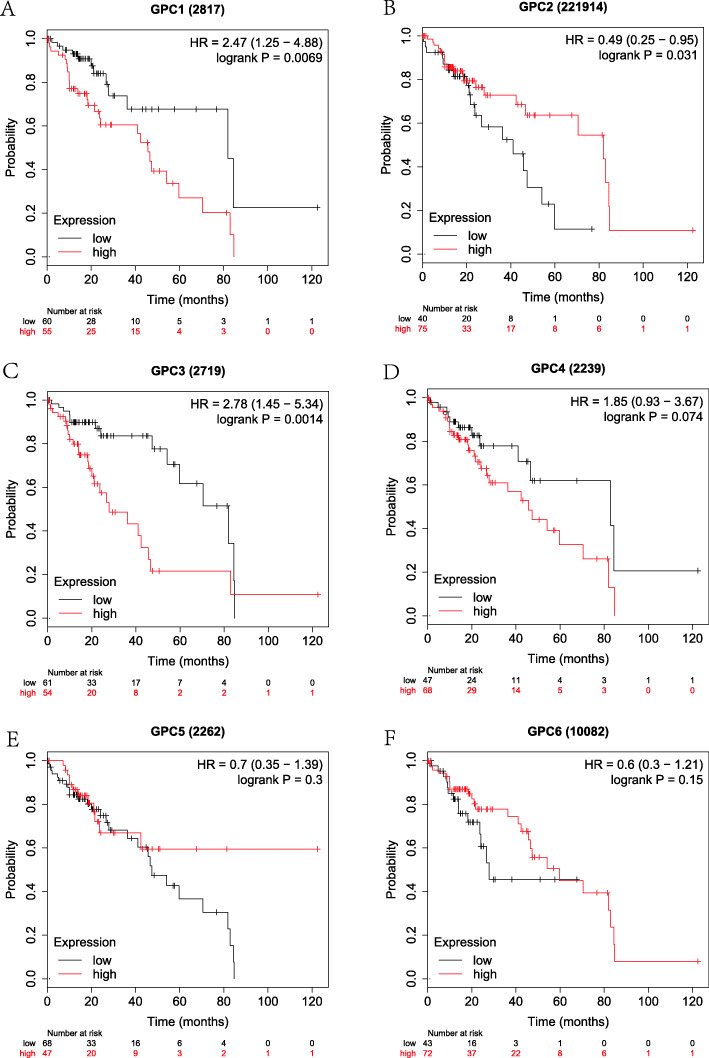
The prognostic value of the mRNA expressions of GPC genes in alcohol consumption HCC patients using KM plotter tool. **a-f**) GPC-1 ~ GPC-6.(*n* = 115)

**Fig. 7:**
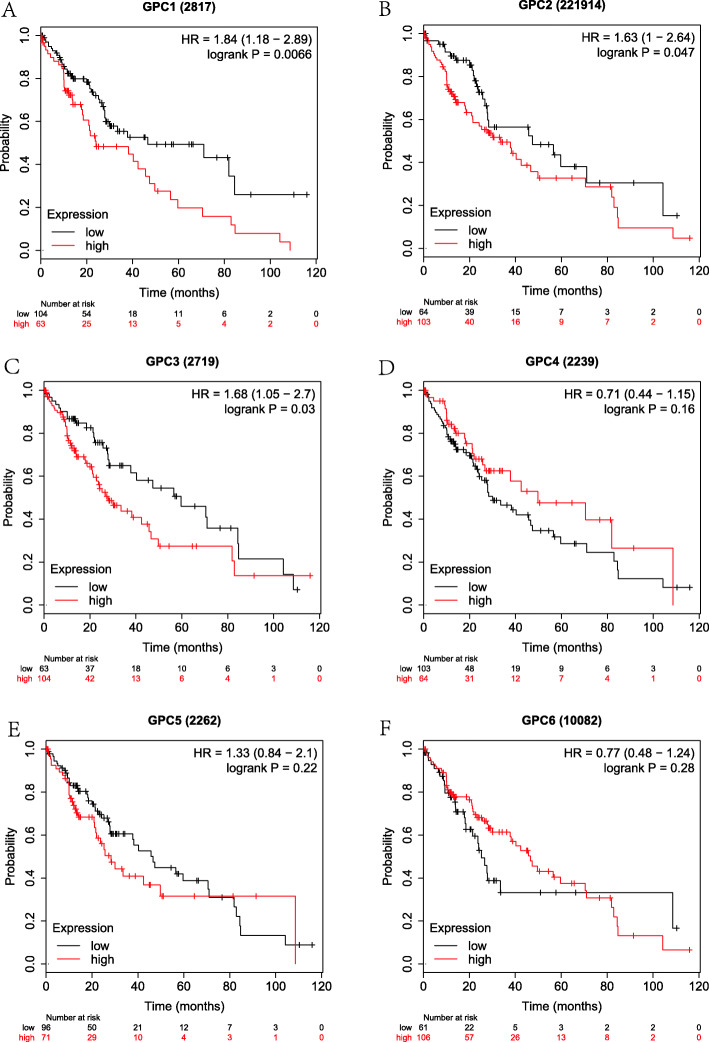
The prognostic value of the mRNA expressions of GPC genes in non-hepatitis virus HCC patients using KM plotter tool. **a**-**f** GPC-1 ~ GPC-6.(*n* = 167)

Firstly, the correlation between the expressions of GPC-1 ~ 6 at the mRNA level and prognosis in HCC patients was investigated using on-line survival analysis database. The Affymetrix ID of GPC-1 in the KM plotter was 2817_at. We found that in overall HCC patients, the up-regulation of GPC-l at the mRNA level was dramatically correlated to a lower OS (HR = 2.03, 95%CI = 1.44–2.87, *P* = 4.1e-05, Fig. 4a). The up-regulation of GPC-l at the mRNA level was remarkably correlated to a lower OS in pathological stage (I ~ III), grade (I ~ III), AJCC_T (I ~ III) and none vascular invasion group (HR = 2.8, 95%CI = 1.48–5.27, *P* = 0.00092; HR = 2.59, 95% CI = 1.08–6.21, *P* = 0.0275; *P* = 0.0397, HR = 1.87, 95%CI = 1.02–3.43; HR = 3.1, 95%CI = 1.1–8.7, *P* = 0.0246; HR = 1.76, 95%CI = 1.04–2.96, *P* = 0.0318; HR = 2.63, 95%CI = 1.42–4.86, *P* = 0.0014; HR = 2.42, 95%CI = 1.32–4.42, *P* = 0.0032; HR = 2.5, 95%CI = 1.1–5.66, *P* = 0.0232; HR = 1.95, 95%CI = 1.04–3.67, *P* = 0.0342; HR = 2.13, 95%CI = 1.26–3.59, *P* = 0.0037, Fig. 9a-d). There were statistically significant differences in the males and Caucasians (HR = 2.46, 95%CI = 1.57–3.85, *P* = 4.5e-5; HR = 1.59, 95%CI = 1.01–2.51, *P* = 0.0434, Supplemental Table 1, 2). Our data also showed that the up-regulation of GPC-l at the mRNA level was remarkably correlated to a lower OS in non-alcohol consumption, non-hepatitis virus group (HR = 2.14, 95%CI = 1.35–3.39, P = 0.001; HR = 1.84, 95%CI = 1.18–2.89, *P* = 0.0066, Figs. 5, 7a) as well as alcohol consumption and hepatitis virus group (HR = 2.47, 95%CI = 1.25–4.88, *P* = 0.0069; HR = 2.38, 95%CI = 1.22–4.62, *P* = 0.0087, Fig. 6, 8a). These results were consistent with the general trend of HCC (*P* < 0.05, Fig. 4a).

The Affymetrix ID of GPC-2 in the KM plotter was 221914_at. The up-regulation of GPC-2 at the mRNA level was not correlated to OS for all HCC patients (HR = 1.36, 95%CI = 0.94–1.96, *P* = 0.1012, Fig. 4b). Figure 9a-d reveals that the pathological factors, including stage, grade, AJCC_T, and vascular invasion, affected the mRNA expression of GPC-2 and prognosis of HCC (*P* > 0.05). There were statistically significant differences in the Asian group (HR = 2.45, 95%CI = 1.33–4.5, *P* = 0.0029, Supplemental Table 2), but no significant difference in the sex group (*P* > 0.05, Supplemental Table 1). The up-regulation of GPC-2 at the mRNA level suggested a worse OS in the non-alcohol consumption group (HR = 2.08, 95% CI = 1.09–3.95, *P* = 0.022, Fig. 5b) and non-hepatitis virus group (HR = 1.63, 95% CI = 1.00–2.64, *P* = 0.047, Fig. 7b). Meanwhile, we analyzed such correlation in the alcohol consumption group and showed that its up-regulation implied a pernicious prognosis (HR = 0.49, 95% CI = 0.25–0.95, *P* = 0.031, Fig. 6b).

Figure 4 shows the prognostic value of GPC-3 (Affymetrix ID:2719_at) expression at the mRNA level. No significant correlation was found between the OS and GPC-3 expression for all HCC patients (*P* > 0.05, Fig. 4c). There was a significant correlation in stage (III), grade (II) and microvascular invasion pathological types (HR = 1.94, 95% CI = 1.08–3.51, *P* = 0.0252; HR = 1.74, 95% CI = 1.05–2.9, *P* = 0.0309; HR = 2.17, 95% CI = 1–4.72, *P* = 0.0442, Fig. 9a-b, d), but no significant difference between sex, race and sorafenib treatment subgroup (*P* > 0.05, Supplemental Table 1, 2, 3). We further analyzed the effects of risk factors on the prognosis of HCC. The up-regulation of GPC-3 at the mRNA level suggested a worse OS in the alcohol consumption group (HR = 2.78, 95% CI = 1.45–5.34, *P* = 0.0014, Fig. 6c) or non-hepatitis virus group (HR = 1.68, 95% CI = 1.05–2.70, *P* = 0.003, Fig. 7c). In contrast, the up-regulation of GPC-3 at the mRNA level was correlated to a better OS in the non-alcohol consumption group (HR = 0.59, 95% CI = 0.37–0.93, *P* = 0.023, Fig. 5c).

**Fig. 8:**
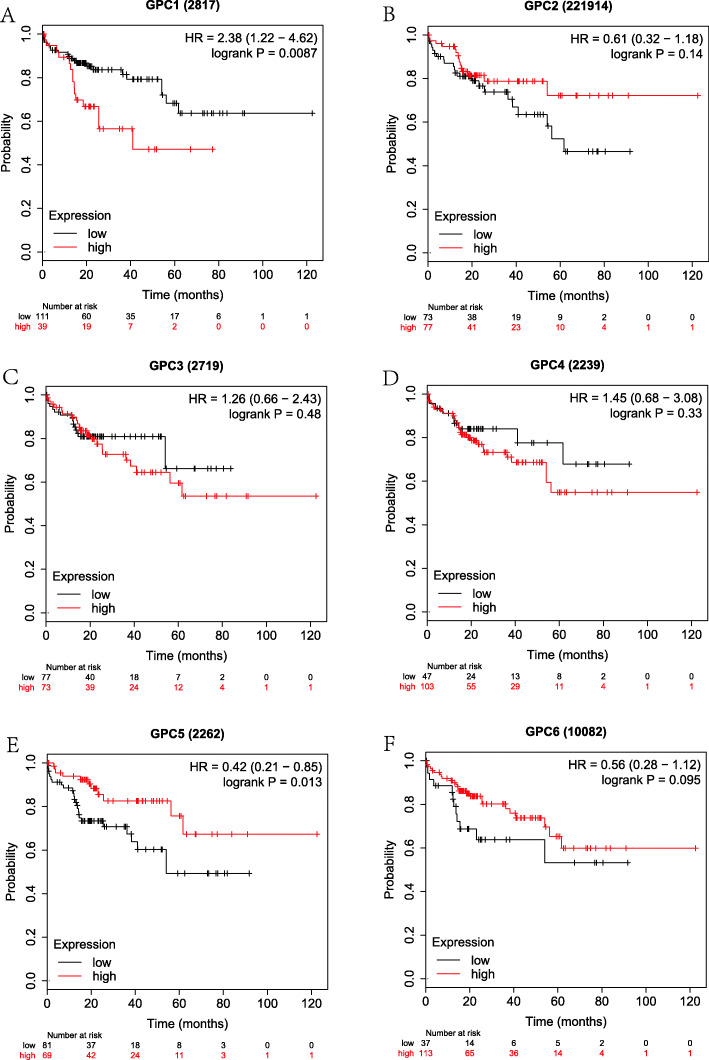
The prognostic value of the mRNA expressions of GPC genes in hepatitis virus HCC patients using KM plotter tool. **a-f** GPC-1 ~ GPC-6.(*n* = 150)

**Fig. 9:**
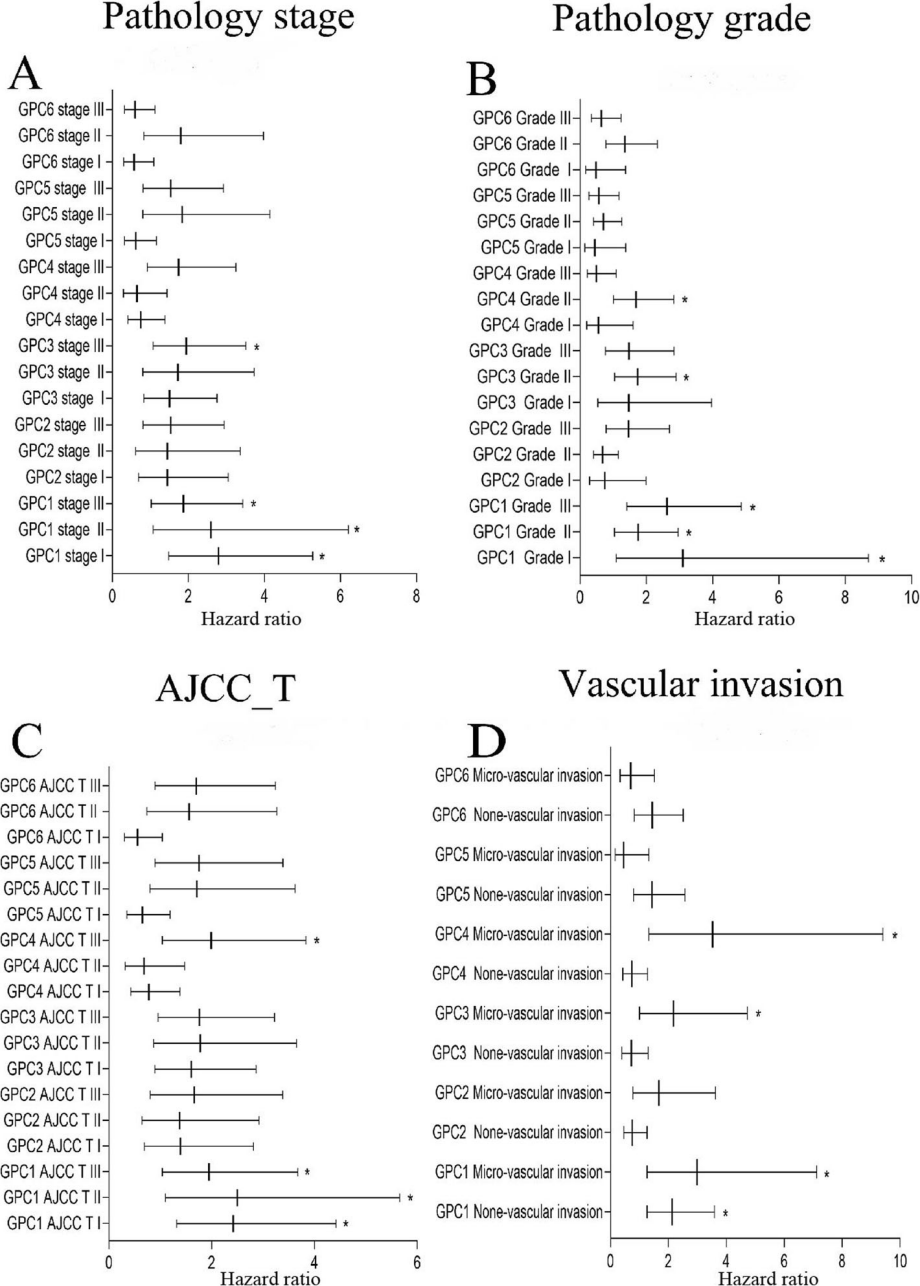
The prognostic value of the mRNA expressions of GPC genes in different pathological factor groups in HCC patients. **a-d** Pathology stage, pathology grade, AJCC_T. Note: *:< 0.05

Subsequently, the effect of GPC-4 (Affymetrix ID:2239_at) was investigated. Figure 4 shows that the expression of GPC-4 exerted no effects on OS for all HCC patients (*P* > 0.05, Fig. 4d). In addition, the down-regulation of GPC-4 at the mRNA level suggested a better OS in the grade (II), AJCC_T (III) subgroup (HR = 1.69, 95% CI = 1.01–2.83, *P* = 0.0435; HR = 1.99, 95% CI = 1.04–3.83, *P* = 0.0343, Fig. 9b-c), whereas it indicated a worse OS in Caucasian HCC patients (HR = 1.56, 95% CI = 0.35–0.91, *P* = 0.0167, Supplemental Table 2). There were also no effects of risk factors, including alcohol consumption and hepatitis virus, on the prognosis of HCC, and no correlation between the expression of GPC-4 at the mRNA level and OS was found in different groups (*P* > 0.05, Figs. 5, 6, 7 and 8 D).

The Affymetrix ID of GPC-5 was 2262_at (Fig. 4e). The up-regulation of GPC-5 at the mRNA level had no relationship with the OS for HCC patients (*P* > 0.05, Fig. 4e), whereas such an up-regulation exhibited a favorable effect on OS in hepatitis virus HCC, sorafenib treatment group (HR = 0.42, 95% CI = 0.21–0.85, *P* = 0.0125, Fig. 8e; HR = 4.56, 95% CI = 0.94–22.27, *P* = 0.0439, Supplemental Table 3). The result also demonstrated that the expression of GPC-5 with female factors might affect the prognosis of HCC (HR = 1.59, 95% CI = 0.91–2.78, *P* = 0.0992, Supplemental Table 1).

We further assessed the prognostic value of GPC-6 expression at the mRNA level (Affymetrix ID:10082_at). The expression of GPC-6 had a significant impact on the OS in Caucasians and sorafenib-treated HCC subgroup (HR = 0.59, 95% CI = 0.36–0.97, *P* = 0.0364; HR = 0.15, 95% CI = 0.04–0.56, *P* = 0.0012, Supplemental Table 2, 3). The data showed that the expression of GPC-6 exerted no effect on the OS of general HCC patients as well as a pathological factor and risk factor subgroup (*P* > 0.05, Fig. 4f, Figs. 5, 6, 7 and 8 F, Fig. 9a-d).

## Discussion

In the present study, we aimed to assess the associations between GPC gene family members and HCC prognosis. We collected the clinical and pathological data of 364 HCC patients from GEO, EGA, and TCGA databases. The data were classified to discuss the prognostic value of GPC family genes by bioinformatics analysis. The study results showed that the expression level of GPC-1 was related to the overall prognosis of HCC. The lower the expression level of GPC-1, the longer the survival time of patients. Among them, GPC-1 was related to the prognosis of HCC in pathological stage, grade, AJCC, vascular invasion, sex, race, risk factors (alcohol consumption and hepatitis) subgroups, and the expression level of GPC-1 was negatively correlated with the OS of HCC. The lower the expression level of GPC-1, the better the prognosis. Besides, the mRNA expressions of GPC-2, 3, 4, 5, and 6 were not related to the overall prognosis of HCC. In the analysis of each subgroup, the mRNA expression of GPC-2 was related to OS with the impact factors of the race (Asian), alcohol consumption, and hepatitis. The mRNA expression of GPC-3 was related to OS in pathological stage (III), grade (II), vascular invasion (micro), sorafenib treatment, alcohol consumption, and hepatitis. The expression level of GPC-4 was related to poor prognosis OS of HCC in grade (II) and AJCC (III) and race (Caucasian and Asian). The expression level of GPC-5 was related to the poor prognosis of OS in sex (female), sorafenib treatment, and hepatitis. The expression level of GPC-6 was related to the poor prognosis of OS in the race (Caucasian) and sorafenib treatment. It could be seen that the significance of GPC family genes in the prognosis of HCC was not the same among different prognostic factors. Based on our findings, we speculated that GPC-1 was stable in predicting the clinical prognosis of each subgroup of HCC, and it could be a good prognostic biomarker. At present, there is no study on the relationship between GPC-1 and HCC prognosis. Our data provided valuable insights into future research and clinical work on HCC. Besides, the predictive effect of GPC-3 on the prognosis of HCC is not consistent among different centers [23–26]. Our results indicated that the expression of GPC-3 was not related to the overall prognosis of HCC, and this result was consistent with Chen IP’s report [27].

GPCs refer to a subset of cell-surface glycoproteins, among which HS glycosaminoglycan chains are covalently attached to a protein core. The GPC family genes are highly conserved across animal species, and they have critical functions in various biological processes [28]. Moreover, GPC genes also play a fundamental role in some biological processes, and they have been considered to be the regulators of several cell signal transduction pathways [29, 30]. The GO and KEGG analyses also showed that GPC genes were mainly involved in Wnt signaling pathway, plasma membrane and planar cell polarity pathway, as well as glycosaminoglycan biosynthetic and catabolic processes. In our study, GGI and PPI networks showed that the GPC genes and other associated genes constructed an intricate network, in which they interacted with each other. Among them, GPC-3 played a core role in the network connection. The results suggested that GPC-3 also played a role in HCC.

GPC-1 is a cell surface HS proteoglycan that is over-expressed in a variety of solid tumors, while its expression is suppressed in most adult normal tissues. GPC-1 acts as a form of co-receptor for a range of signaling molecules, affecting signaling pathways, including Wnt, Hedgehog, TGF-β, and fibroblast growth factor [31]. GPC-1 is an important clinical biomarker involved in the process of cancer onset, and it can be used as an important indicator of disease prognoses, such as breast cancer, lung cancer, colorectal cancer, glioma, pancreatic cancer, and esophageal cancer [32]. Matsuda K et al. have found that GPC-1 may play a pivotal role in the ability of breast cancer cells to exhibit a mitogenic response to multiple heparin-binding growth factors and contribute to disease progression in this malignancy [33]. Qian JY et al. have reported that GPC-1 in exosomes is identified as an early diagnostic biomarker for pancreatic adenocarcinoma, and the presence of GPC-1 in extracellular vesicles can serve as a predictor of RIAC outcome for patients with APC mutation [34]. Although GPC-1 can be used as a diagnostic marker and a prognostic indicator in many cancers, the clinical value of GPC-1 in the diagnosis and prognosis of HCC has not been reported [35]. Our study showed that the GPC-1 could be a good potential biomarker in HCC prognosis.

As an oncofetal glycoprotein, GPC-3 binds to the cell surface through a glycophosphatidylinositol anchor. To date, GPC-3 is the most well-documented GPC gene in its family in different cancers, including HCC, ovarian clear cell carcinoma, melanoma, squamous cell carcinoma of the lung, hepatoblastoma, nephroblastoma (Wilms tumor), yolk sac tumor, and some pediatric cancers [36]. GPC-3 is expressed in over 70% of HCC cases [37]. Capurro MI et al. have found that GPC-3 can trigger canonical Wnt signaling, leading to the accelerated growth of HCC [38]. Besides its role as a biomarker, more and more attention has been paid to GPC-3 as a new therapeutic target molecule, and clinical trials targeting GPC-3 are in progress [39]. Ortiz MV et al. have found that GPC-3-targeted immunotherapy is a key pediatric-specific consideration [40]. Jeon Y et al. have concluded that GPC-3 expression is more frequently correlated to HCC with aggressive features in South Koreans [41]. Zhang J et al. have revealed that the over-expression of GPC-3 can predict a poor OS in HCC patients. Although GPC-3 has been established as a vital prognostic index for HCC, the results remain controversial [23–26]. According to our research, the results showed that the expression of GPC-3 was increased in HCC tissues, but GPC-3 could not predict the overall prognosis of HCC. Chen IP et al. have studied 55 patients with early HCC who undergo initial hepatectomy between 1995 and 2010. They find that the GPC-3 expression is not significantly correlated with OS [42]. This outcome is consistent with our result. The controversial conclusions might be attributed to the clinical cases. Although there are certain standards for data collection, some differences still exist in the data collection methods of each center. Each experimental sample contains different pathological, clinical and risk factors, leading to the biased results. Therefore, the data uniformity could not be guaranteed. Our data were derived from the public dataset and different centers. Information obtained from multiple centers can avoid error as much as possible.

A recent study has revealed that GPC-2 can positively regulate Wnt signaling in neuroblastoma, as silencing of GPC-2 inactivates Wnt/b-catenin signaling and reduces the expressions of target genes [43]. As one of several mRNA transcripts, GPC-2 is highly expressed in several childhood cancers. Orentas RJ et al. have revealed that GPC-2 has a critical function in neurodevelopment, childhood cancer, and prostate cancer [44]. Xu N et al. have reported that the GPC-2 plays a fundamental role in the progression and metastasis of prostate cancer and thus can be used as a candidate treatment target and a latent prognostic biomarker [45]. Bosse KR has revealed that GPC-2 is a suitable tumor antigen in neuroblastoma [46]. Although HCC is concerned, the diagnosis and prognosis associated with the GPC-2 expression at the mRNA level have not been reported. In our study, the result also revealed that the expression of GPC-2 was not related to the overall prognosis of HCC.

GPC-5 is a member of the GPC family, which is capable of binding to the external surface of the plasma membrane [47]. GPC5 is a critical regulator of morphogens and growth factors during development [48]. The GPC5 gene is located on chromosome 13q31.3, a region that is frequently mutated in many types of cancers [49]. GPC-5 has been reported as a tumor suppressor gene in many cancers, such as pancreatic cancer, glioma, endometrial cancer, prostate cancer, and lung adenocarcinoma. Liu T et al. have shown that miR-709 enhances the invasion of HCC cells by mediating the GPC-5 expression [50]. Hong X et al. have reported that GPC-5 plays a tumor-suppressive role in glioma and indicated that the inhibition of miR-301b suppresses the proliferation and invasion of glioma cells by increasing the expression of GPC-5 [50, 51]. Our study revealed that the expression level of GPC-5 was not related to the prognosis of OS in overall HCC patients.

Compared with GPC-1, GPC-2, GPC-3, and GPC-5, few studies have investigated the mechanism and prognostic significance of GPC-4 and GPC-6 expressions at the mRNA level in malignant tumors, and no related reports have been found in HCC. GPC-4 is a novel adipomyokine that enhances insulin signaling. Ussar et al. have initially shown that GPC-4, as a novel adipokine, is released from cells and adipose tissue of mouse explants, and this circulating GPC-4 interacts with the insulin receptor and enhances insulin receptor signaling and insulin sensitivity [49]. Cao J et al. have demonstrated that GPC-4 participates in 5-fluorouracil (5-FU) resistance and pancreatic cancer stemness [52]. Zhao D et al. have demonstrated that the T allele of GPC-4 may signify a risk factor for Epstein-Barr virus-associated and negative gastric carcinoma [53]. As the newest member of the family, GPC-6 is most homologous to GPC4 and ubiquitously expressed [54]. Karapetsas A et al. have reported that GPC-6 may function as a predictive indicator of CD8+ T-lymphocyte infiltration and a favorable prognostic index in early-stage ovarian cancer, showing significant clinical value in diagnosis, prognosis, and tumor immunobattling [55]. Fan Cet al. have demonstrated that GPC-6 promotes proliferation, migration, and invasion of tumor cells in nasopharyngeal carcinoma [56]. Our study also suggested that there was no difference in the expressions of GPC-4 and GPC-6 in normal liver and HCC, and these two genes had no predictive effect on the overall prognosis of HCC.

From our data graph, we could also see some interesting perspectives. Firstly, in terms of race, the prognosis of the Caucasian was related to most GPC family genes, while the prognosis of the Asian was only related to the expression of GPC-2. There were significant differences in the prognostic significance of GPC genes among different races. Secondly, for pathological factors, including stage, grade, AJCC, and vascular invasion, the higher the pathological grade and vascular invasiveness, the lower the expression levels of GPC family genes. The expression levels of GPC family genes could be used as an indicator of poor pathological prognosis, which was also consistent with common sense. Thirdly, in the risk factor (alcohol consumption and hepatitis) subgroup, the expression levels of GPC-1, 2, and 3 in the hepatitis group were related to the poor prognosis of HCC. According to our study, the reason for this opposite conclusion might be attributed to that the risk factor of drinking alcohol affected the expression levels of GPC-2 and GPC-3. The incidence, diagnosis, treatment, and medical status of HCC in each region were different, leading to the different prognosis of HCC. Consequently, the overall relationship between GPC genes and prognosis obtained from the public database would show some differences. We speculated that the pathology, patients, and risk factors might affect the GPC family’s prognostic value in HCC.

There were some limitations in this study that should be addressed. First, the study cohort was relatively small. Thus, larger studies are required to verify these findings. Besides, further studies from multiple centers with patients of various races are needed. Finally, our study was mainly based on the online website, which generates univariate analysis. Based on the present study, further explorations using multivariate analysis will aim to determine independent factors and potential molecular mechanisms. To address these issues, we are planning well-designed functional verification studies, including in vitro and in vivo models, shortly.

## Conclusions

Collectively, this study aimed to assess the associations between HCC prognosis and the expression patterns of GPC family members. Our study found that GPC-3 was dysregulated in HCC compared with paracancerous tissues. Furthermore, the up-regulation of GPC-1 at the mRNA level was dramatically correlated to the reduced OS for overall HCC patients. Besides, the pathology, patients, and risk factors might affect the expressions of GPC genes and the clinical stage of HCC. Therefore, GPC-1 was a potentially prognostic biomarker for HCC. However, our findings still need to be further validated, and the prognostic values of other GPC genes still need to be prospectively confirmed in a larger number of patients.

## Supplementary Information

**Additional file 1: Supplemental Table 1.** The prognostic value of the mRNA expressions of GPC genes in different sex HCC patients.

**Additional file 2: Supplemental Table 2.** The prognostic value of the mRNA expressions of GPC genes in different race HCC patients.

**Additional file 3: Supplemental Table 3.** The prognostic value of the mRNA expressions of GPC genes in sorafenib treatment HCC patients.

## Data Availability

All analyzed data are included in this published article. The original data are available upon reasonable request to the corresponding author. The public access of links to all databases are open and available to all the visitors. DAVID (https://david.ncifcrf.gov/home.jsp); GeneMANIA (http://www.genemania.org/); STRING (https://string-db.org/); GEPIA (http://gepia.cancer-pku.cn/index.html); Kaplan-Meier plotter (http://kmplot.com/analysis/index.php?p=service&cancer=liver_rnaseq);

## Abbreviations

HCC: Hepatocellular carcinoma
GPC: Glypican
GPCs: Glypicans
HS: Heparan sulfate
GO: Gene Ontology
KEGG: Kyoto Encyclopedia of Genes and Genomes
GGI: Gene-gene interaction
PPI: Protein-protein interaction
GEPIA: Gene Expression Profiling Interactive Analysis
OS: Overall survival
GEO: Gene Expression Omnibus
EGA: European Genome-phenome Archive
TCGA: The Cancer Genome Atlas
HR: Hazard ratio
CI: Confidence intervals
5-FU: 5-fluorouracil
