# Supplementary material for: Distinct diagnostic and prognostic values of Glypicans gene expression in patients with hepatocellular carcinoma
Source: BMC Cancer. 2021 Apr 26;21:462. doi: 10.1186/s12885-021-08104-z (PMC8073913; doi:10.1186/s12885-021-08104-z)
Supplement: Supplementary file 1 — Additional file 1: Supplemental Table 1. The prognostic value of the mRNA expressions of GPC genes in different sex HCC patients. [file 12885_2021_8104_MOESM1_ESM.docx]

Supplemental Table 1: The prognostic value of the mRNA expressions of GPC genes in different sex HCC patients.

| Gene | Sex | Cases | HR (95%CI) | P value |
| --- | --- | --- | --- | --- |
| GPC1 | male | 246 | 2.46(1.57-3.85) | 4.5e-5 |
|  | female | 118 | 1.47(0.84-2.57) | 0.1703 |
| GPC2 | male | 246 | 1.54(0.95-2.5) | 0.0786 |
|  | female | 118 | 1.51(0.76-3.03) | 0.2388 |
| GPC3 | male | 246 | 1.55(0.99-2.41) | 0.0519 |
|  | female | 118 | 1.38(0.77-2.47) | 0.2796 |
| GPC4 | male | 246 | 1.48(0.95-2.32) | 0.0833 |
|  | female | 118 | 0.66(0.38-1.15) | 0.1422 |
| GPC5 | male | 246 | 0.53(0.32-0.86) | 0.0098 |
|  | female | 118 | 1.59(0.91-2.78) | 0.0992 |
| GPC6 | male | 246 | 0.68(0.44-1.07) | 0.0949 |
|  | female | 118 | 0.68(0.36-1.29) | 0.2359 |

Notes: GPC, glypican; HCC, hepatocellular carcinoma; HR, hazard ratio; CI, confidence interval.
