# Supplementary material for: Distinct diagnostic and prognostic values of Glypicans gene expression in patients with hepatocellular carcinoma
Source: BMC Cancer. 2021 Apr 26;21:462. doi: 10.1186/s12885-021-08104-z (PMC8073913; doi:10.1186/s12885-021-08104-z)
Supplement: Supplementary file 2 — Additional file 2: Supplemental Table 2. The prognostic value of the mRNA expressions of GPC genes in different race HCC patients. [file 12885_2021_8104_MOESM2_ESM.docx]

Supplemental Table 2: The prognostic value of the mRNA expressions of GPC genes in different race HCC patients.

| Gene | Race | Cases | HR (95%CI) | P value |
| --- | --- | --- | --- | --- |
| GPC1 | white | 181 | 1.59(1.01-2.51) | 0.0434 |
|  | Black/african american | 17 | - | - |
|  | asian | 155 | 2.55(1.33-4.88) | 0.0033 |
| GPC2 | white | 181 | 0.65(0.4-1.04) | 0.0708 |
|  | Black/african american | 17 | - | - |
|  | asian | 155 | 2.45(1.33-4.5) | 0.0029 |
| GPC3 | white | 181 | 1.65(0.95-2.85) | 0.0700 |
|  | Black/african american | 17 | - | - |
|  | asian | 155 | 1.47(0.82-2.66) | 0.195 |
| GPC4 | white | 181 | 1.56(0.35-0.91) | 0.0167 |
|  | Black/african american | 17 | - | - |
|  | asian | 155 | 2.45(1.26-4.76) | 0.0064 |
| GPC5 | white | 181 | 0.68(0.39-1.19) | 0.173 |
|  | Black/african american | 17 | - | - |
|  | asian | 155 | 0.7(0.37-1.33) | 0.274 |
| GPC6 | white | 181 | 0.59(0.36-0.97) | 0.0364 |
|  | Black/african american | 17 | - | - |
|  | asian | 155 | 0.59(0.31-1.1) | 0.0918 |

Notes: GPC, glypican; HCC, hepatocellular carcinoma; HR, hazard ratio; CI, confidence interval.
