# Supplementary material for: Distinct diagnostic and prognostic values of Glypicans gene expression in patients with hepatocellular carcinoma
Source: BMC Cancer. 2021 Apr 26;21:462. doi: 10.1186/s12885-021-08104-z (PMC8073913; doi:10.1186/s12885-021-08104-z)
Supplement: Supplementary file 3 — Additional file 3: Supplemental Table 3. The prognostic value of the mRNA expressions of GPC genes in sorafenib treatment HCC patients. [file 12885_2021_8104_MOESM3_ESM.docx]

Supplemental Table 3: The prognostic value of the mRNA expressions of GPC genes in sorafenib treatment HCC patients.

| Gene | Sorafenib | Cases | HR (95%CI) | P value |
| --- | --- | --- | --- | --- |
| GPC1 | treatment | 29 | 1.76(0.59-5.28) | 0.3051 |
| GPC2 | treatment | 29 | 0.41(0.12-1.37) | 0.1348 |
| GPC3 | treatment | 29 | 3.37(0.91-12.49) | 0.0534 |
| GPC4 | treatment | 29 | 1.97(0.53-7.38) | 0.3045 |
| GPC5 | treatment | 29 | 4.56(0.94-22.27) | 0.0439 |
| GPC6 | treatment | 29 | 0.15(0.04-0.56) | 0.0012 |

Notes: GPC, glypican; HCC, hepatocellular carcinoma; HR, hazard ratio; CI, confidence interval.
